# Delayed Crosslinking and Plugging Performance of Polyacrylamide Gel Using CaCl_2_-Tolerant Delayed-Release Crosslinker in High-Calcium Medium

**DOI:** 10.3390/gels12080725

**Published:** 2026-08-14

**Authors:** Huajie Liu, Zhiwei Tao, Theis I. Solling, Sergei E. Chernyshov, Huanan Zhang, Liming Zhang, Dmitriy A. Martyushev

**Affiliations:** 1 School of Petroleum Engineering, China University of Petroleum (East China), Qingdao 266580, China; 2 State Key Laboratory of Deep Oil and Gas, China University of Petroleum (East China), Qingdao 266580, China; 3 Shandong Key Laboratory of Oil and Gas Field Chemistry, School of Petroleum Engineering, China University of Petroleum (East China), Qingdao 266580, China; 4 School of Petroleum Engineering, King Fahd University of Petroleum and Minerals-KFUPM, Dammam 31433, Saudi Arabia; 5 Oil and Gas Technologies Department, Perm National Research Polytechnic University, Perm 614990, Russia

**Keywords:** polyacrylamide gel, hydroxyaluminum, CaCl_2_-tolerant, delayed-release crosslinker, intelligent gel

## Abstract

Lost circulation is a major technical bottleneck restricting safe and efficient while-drilling plugging operations. Polyacrylamide gel has become a widely used plugging material in drilling engineering. Unlike rigid, cement-like plugging materials, the gel system formed in this study does not develop a hardened, consolidated structure capable of anchoring or binding the drill bit during subsequent drilling operations, thereby eliminating the risk of bit-sticking. Nevertheless, the gel possesses sufficient elastic (viscoelastic) structural strength—reflected in its storage modulus (G′)—to effectively resist deformation and displacement under differential pressure, thereby providing reliable fracture-sealing performance, which effectively prevents pipe-sticking risks. However, high-concentration PAM molecular chains easily stretch and entangle in aqueous solution, triggering an abnormal increase in initial viscosity and poor pumpability. Although Ca^2+^ can inhibit the premature water absorption and thickening of PAM to maintain system fluidity, an excessively high Ca^2+^ concentration will suppress the hydrolysis of Al^3+^ and hinder the formation of hydroxyaluminum—the key crosslinking component of the gel system. To solve the above contradiction, a CaCl_2_-tolerant delayed-release crosslinker was synthesized. ZnO was selected as a carrier to adsorb and immobilize polynuclear hydroxyaluminum complexes hydrolyzed from an inorganic aluminum crosslinker at 70 °C, realizing the controlled delayed release of the crosslinker. The microstructures and chemical bonding were characterized by SEM elemental mapping, FT-IR and ^27^Al MAS NMR. The results confirm that abundant aluminum species are uniformly loaded on the ZnO surface to form stable Zn–O–Al covalent bonds, and the loaded aluminum exists mainly in the form of hydroxyaluminum. With increasing temperature, the Zn–O–Al bonds gradually break and slowly release hydroxyaluminum species. A novel delayed crosslinking gel system was ultimately optimized, composed of 6% CaCl_2_, 10.4% PAM and 3% ZnO loaded with polynuclear hydroxyaluminum. The system exhibits excellent delayed gelation behavior, with a fluidity loss time longer than 120 min and a gelation time over 200 min. It maintains favorable fluidity within 30–90 °C, and the formed gel shows a stable elastic modulus (G′) and viscous modulus (G″). Moreover, the system achieves a plugging rate of more than 90% and a breakthrough pressure above 5 MPa, demonstrating superior comprehensive plugging performance for while-drilling plugging applications.

## 1. Introduction

Lost circulation not only consumes massive amounts of drilling fluid, but also prolongs drilling time. Improper handling may also trigger a series of complex drilling accidents, including borehole collapse, blowout and pipe-sticking. These factors impair the rate of penetration and incur substantial economic losses, thus forming a major technical bottleneck restricting progress in oil and gas exploration and development [1,2,3,4]. Different from the traditional shut-in plugging method that requires stopping drilling, while-drilling plugging can perform plugging construction synchronously without interrupting drilling operations. It avoids the frequent tripping of drilling tools, maintains continuous drilling work, and greatly improves operational efficiency. This technology can effectively seal micro-fractures and natural leakage channels in advance, stabilize the wellbore wall in real time, and prevent secondary accidents such as wellbore collapse and tool-sticking caused by long-term shutdown. Owing to its outstanding formation adaptability, it is suitable for complex harsh strata such as fractured reservoirs and high-calcium saline formations, and has evolved into a critical technical means to address frequent lost circulation and guarantee safe drilling [3,4]. At present, mainstream lost circulation control methods for while-drilling operations are mainly divided into four categories: inorganic material plugging, cement plugging, physical temporary plugging, and organic polymer material plugging. Each method has different technical characteristics and applicable scenarios, yet most cannot satisfy the safety and adaptability requirements of while-drilling plugging: inorganic material plugging features high rigid strength and low cost, but suffers from poor pumpability and difficulty in entering tiny loss channels, failing to meet the continuous pumping demand during drilling [5]; cement plugging can form a high-strength consolidation body, but it tends to solidify rapidly and uncontrollably, which easily leads to bit-sticking and borehole blockage, making it totally unsuitable for while-drilling plugging; physical temporary plugging boasts fast construction and easy unblocking, yet is plagued by shortcomings such as weak plugging tightness and poor erosion resistance, rendering it unable to maintain stable plugging during drilling operations [6,7]; organic gel plugging, with its unique advantages of excellent rheology, a deep migration capability and strong sealing performance, has become a core technology for controlling complex lost circulation zones such as shallow micro-fractures and fracture–vug composite formations, and presents prominent application potential in while-drilling plugging [8]. However, most conventional gel plugging systems adopt an instantaneous gelation mode. They tend to cause pump-plugging and drill-sticking due to premature crosslinking, or suffer from gel erosion and loss by formation fluids as a result of delayed crosslinking, which severely restricts their field adaptability for while-drilling operations [9,10]. Delayed crosslinking technology enables low-viscosity delivery of the gel in the wellbore and its deep migration into the reservoir, allowing crosslinking to occur precisely at the targeted leakage zones. This effectively prevents wellbore-plugging and near-wellbore reservoir damage caused by premature crosslinking, making it the optimal technical solution for safe and efficient while-drilling lost circulation control [11,12].

At present, research on delayed crosslinking technology mainly focuses on three aspects: reaction conditions, crosslinking agents, and polymers. Studies on reaction conditions focus primarily on pH and temperature. Novel regulation strategies such as temperature, pH responsiveness and supramolecular host–guest interactions enable the precise and intelligent triggering of crosslinking reactions and are environmentally friendly. However, restricted by preparation costs and applicable temperature ranges, they are in the stage of optimization and improvement before industrial application [13,14]. With regard to crosslinkers, the main approaches include ligand regulation, crosslinker coating and sustained-release, and redox regulation. Among them, the ligand regulation method shields the active sites of metal crosslinking ions such as Zr^4+^ and Cr^3+^ through complexation [15,16]. Under formation temperature and pressure conditions, decomplexation occurs to release active ions and achieve delayed crosslinking. Featuring an excellent delay effect, strong temperature resistance and moderate cost, it is the most widely applied technology in oilfield operations. However, its crosslinking performance is significantly affected by water salinity and system pH [17]. The crosslinker coating and sustained-release method encapsulates crosslinkers in carriers such as polymers and inorganic porous materials via physical or chemical approaches to achieve controlled-rate release. It features high precision in regulating crosslinking timing and a wide range of applicability. However, its preparation process is relatively complex, and the industrial application cost of some microcapsule/microsphere-coating systems is relatively high [18,19]. The redox regulation method is mainly applied to chromium-based crosslinkers. By controlling the reaction rate between reducing agents and Cr^6+^ [20], it realizes the gradual conversion of Cr^6+^ into crosslinking-active Cr^3+^, enabling a long-duration delay of 0.5–24 h and adapting well to high-temperature and high-salinity reservoir conditions. However, due to the toxicity of Cr^6+^, this method entails significant environmental risks in practical applications.

For polymers, the reactivity between polymers and crosslinkers is mainly reduced by means of hydrophobic modification, the degradable protection of crosslinking active sites, or molecular chain branching modification [21]. Such modified polymers feature good compatibility with gel systems and high stability after gelation. Nevertheless, the chemical modification process is cumbersome, resulting in relatively high costs for large-scale production and application. For polyacrylamide (PAM), a high polymer concentration results in high gel strength, but tends to cause premature water absorption and viscosity increases in the polymer. Conversely, a low polymer concentration allows for a controllable crosslinking time, yet yields relatively weak gel strength [22]. Therefore, in practical industrial applications, there is a demand for a delayed crosslinking method that allows for the use of high-hydrolysis-degree PAM without causing premature water absorption and viscosity increase in the polymer, as well as featuring a controllable crosslinking time. Studies have shown that with increasing salinity, the crosslinking time of the gel is significantly prolonged, and the hydration capacity of Ca^2+^ is stronger than that of Na^+^ [23], This endows Ca^2+^ with a stronger competitive water absorption capacity compared to polymer molecules, leading to a more significant reduction in the hydration ability of the polymer chains and more severe chain-coiling. When Ca^2+^ concentration reaches a certain threshold, the gel system can maintain long-term fluidity, but the final gel completely loses its strength [24]. Therefore, a substance needs to be added to the gel system to scavenge calcium ions. Common reagents include sodium carbonate, sodium bicarbonate, and chelating agents such as sodium tartrate and sodium citrate. Although these substances can directly react with calcium ions, they tend to cause excessively rapid crosslinking.

This study found that the ability of the system to maintain fluidity for a long time under a high calcium ion concentration is partly due to polymer molecular-coiling, and partly because the high concentration of Ca^2+^ inhibits the hydrolysis of metal ion crosslinker monomers, resulting in the loss of crosslinking ability. When a non-ionic crosslinker is used, the system can still achieve rapid crosslinking. Therefore, in aqueous solutions with a high calcium ion concentration, this paper proposes a method of adsorbing and loading polynuclear hydroxyaluminum using ZnO. ZnO can control the release rate of polynuclear hydroxyaluminum, thereby delaying the crosslinking time between polynuclear hydroxyaluminum and PAM. At the same time, it prevents Al^3+^ from failing to hydrolyze into polynuclear hydroxyaluminum due to the presence of calcium ions. This method can satisfy the use of high-polymer-concentration polyacrylamide while preventing premature water absorption and viscosity increase in PAM, with a controllable gelation time. Thus, a delayed gelation system for high-concentration polyacrylamide is proposed. In this study, quartz-sand packs of varying mesh size were used as a laboratory-scale proxy for fracture–aperture plugging behavior, enabling systematic and reproducible evaluation of the gel plugging strength across a range of pore/fracture-equivalent opening sizes. Validation under conditions more representative of field fractures—including fractured-core or fracture-slot displacement, drilling-fluid contamination, and a range of aging times—is identified as necessary follow-up work and is discussed. It is worth noting that, alongside inorganic carrier-based systems such as ZnO, growing industry and regulatory emphasis on environmental sustainability has spurred increasing research interest in green and biodegradable alternatives for drilling fluid additives. Among these, cellulose nanofibers (CNF) and other nanocellulose-based materials have been explored as rheology modifiers, crosslinking carriers, and reinforcing/plugging agents in gel and drilling fluid systems, owing to their renewable origin, biodegradability, and favorable surface chemistry [25]. These bio-based materials present promising potential as either alternative or complementary carriers to inorganic materials in lost-circulation control applications. However, challenges remain regarding their thermal stability under high-temperature downhole conditions and their compatibility with high-salinity formation fluids, which currently limit their direct application in deep or complex well scenarios [26]. In contrast, ZnO, as an inorganic carrier, offers superior thermal stability and controllable release performance under the high-temperature, high-salinity conditions targeted in this study. Nevertheless, the environmental profile of ZnO-based systems warrants attention, and future work could explore hybrid or bio-based carrier strategies that combine the performance advantages of inorganic materials with the sustainability benefits of nanocellulose-based systems. In this study, quartz–sand packs of varying mesh size were used as a laboratory-scale proxy for fracture–aperture plugging behavior, enabling systematic and reproducible evaluation of the gel plugging strength across a range of pore/fracture-equivalent opening sizes. Validation under conditions more representative of field fractures—including fractured-core or fracture-slot displacement, drilling-fluid contamination, and a range of aging times—is identified as necessary follow-up work and is discussed in Section 2.4.2.

## 2. Results and Discussion

### 2.1. Effect of CaCl_2_ Concentration on Crosslinking System

#### 2.1.1. Effect of CaCl_2_ Concentration on Al Crosslinker

(1) Al^3+^(aq) + 3H_2_O(l)⇌[Al(H_2_O)_6_]^3+^

(2) pAl^3+^ + qH_2_O⇌Alp(OH)q^(3p – q)^ + qH^+^

where the exponent (3p – q) represents the net positive charge of the polynuclear hydroxoaluminum species. Taking the Keggin-type Al_13_ polycation ([AlO_4_Al_12_(OH)_24_(H_2_O)_12_]^7+^) as a representative example, as discussed in this section, when *p* = 13 and q = 32, the net charge is 3(13) – 32 = +7, i.e., Al_13_^7+^, which validates the charge and mass balance of Equation (2).

Calcium ions do not directly participate in the hydrolysis reaction of aluminum ions. Instead, they regulate the hydrolysis equilibrium, reaction rate and product morphology of aluminum ions through indirect physical and chemical effects, and this influence exhibits a concentration threshold effect. A low–medium concentration of Ca^2+^ (<0.3 mol/L) increases the ionic strength of the system, reduces the activity of Al^3+^ and hydrolytically generated H^+^, and drives the hydrolysis equilibrium to shift right. It also compresses the electrical double layer of hydroxyaluminum species, weakens their electrostatic repulsion, and accelerates the polymerization of oligomers into highly active polynuclear hydroxyaluminum with Al_13_ as the core, thus promoting hydrolysis. In contrast, a high concentration of Ca^2+^ (>0.3 mol/L) reduces the hydration coordination number of Al^3+^ by competing for free water molecules with aluminum ions, drastically lowers the water activity of the system, shifts the hydrolysis equilibrium to the left and inhibits the initial hydrolysis of Al^3+^. Furthermore, it forms a dense ionic atmosphere on the surface of hydroxyaluminum to shield positive charges, prevents the polymerization into active polynuclear species via hydroxyl bridge bonds, and even generates inactive Ca–Al–OH heteronuclear complexes. Eventually, aluminum in the system mainly exists in the form of inactive monomers [27,28].

**Figure 1:**
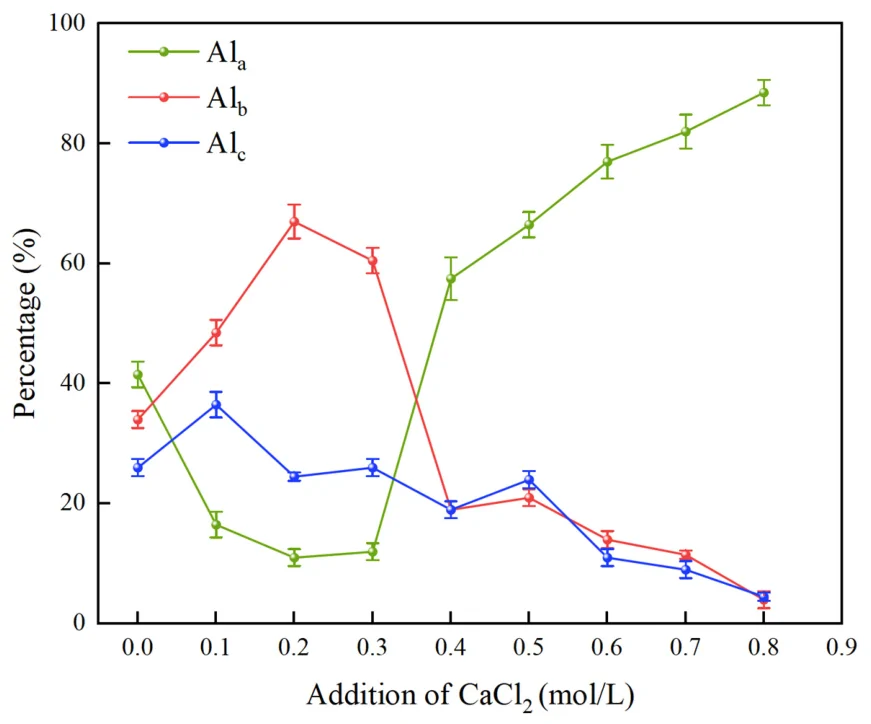
Percentage of hydrolyzed aluminum species versus CaCl_2_ concentration.

**Figure 2:**
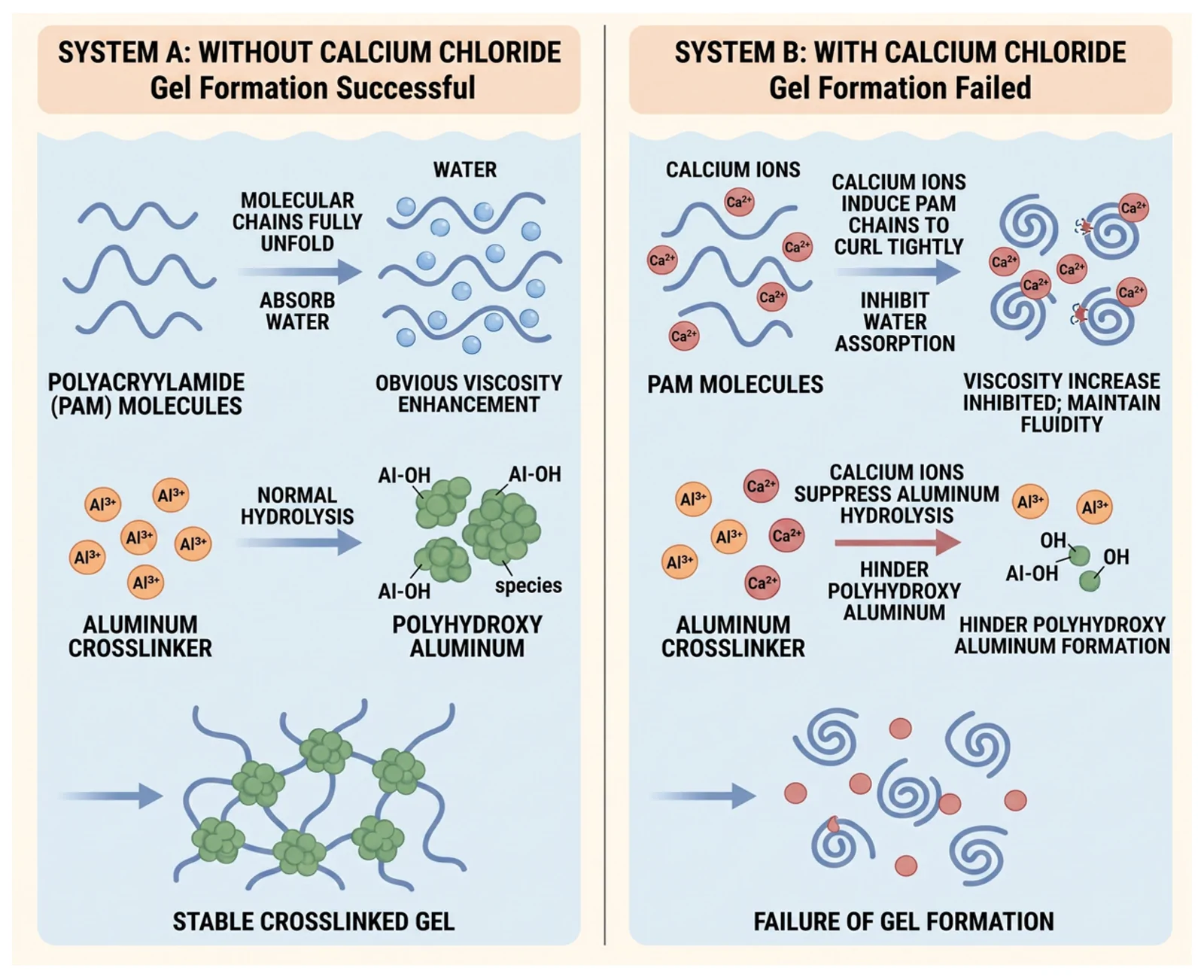
Effect of CaCl_2_ on PAM-AS crosslinked gel formation.

The AS was added to CaCl_2_ solutions of different concentrations, and an aqueous solution of the AS was prepared as the control group. The content change in Alb (polynuclear hydroxyaluminum represented by Al_13_) in the solution was determined by Al–Ferron timed complexation colorimetry [29,30]. The principle of classifying the hydrolytic polymerization species of aluminum by Al–Ferron timed complexation colorimetry is based on the difference in the reaction rate between the chromogenic agent Ferron and hydrolyzed aluminum complexes. The various species in the hydrolyzed aluminum solution are divided into three categories, as shown in Figure 1: Al_a_: mononuclear species, with the fraction reacting instantaneously with Ferron (reaction time: 0–1 min); Al_b_: medium-polymerized species, with the fraction reacting slowly with Ferron (i.e., highly active polynuclear hydroxyaluminum), where the reaction kinetics follows a pseudo-first-order reaction (reaction time < 120 min); Al_c_: generally hydrolytic polymerized macromolecules or colloidal polymers that react very slowly or barely with Ferron (reaction time > 120 min). The content and proportion of each species can be calculated based on the known total aluminum concentration (Al_T_). Figure 2 shows the effect of CaCl_2_ on a Pam/aluminum crosslinked gel formation.

(3) Al_T_ = Al_a_ + Al_b_ + Al_c_

#### 2.1.2. Effect of CaCl_2_ Concentration on Time to Loss of Fluidity

**Figure 3:**
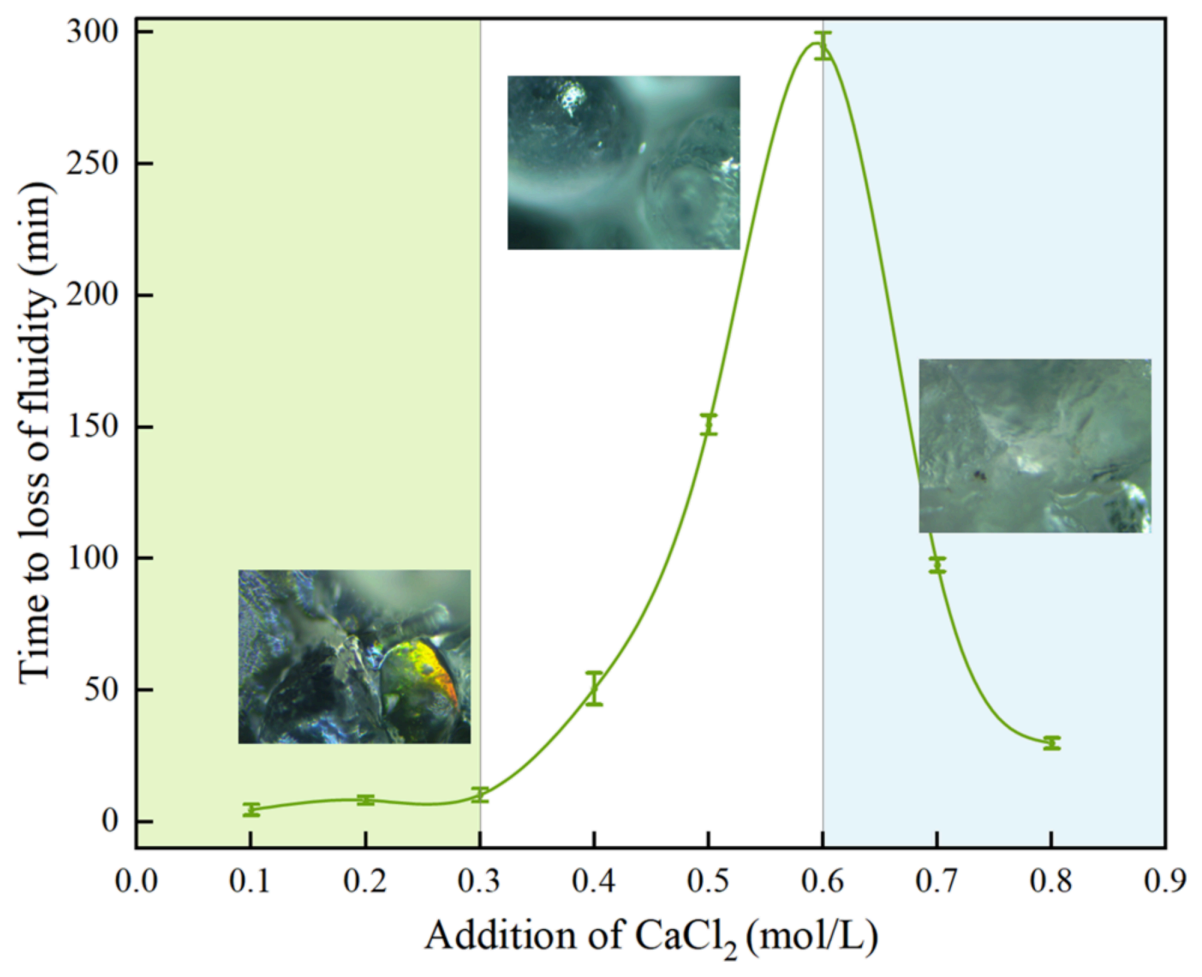
Time-to-loss and time-to-fluidity of PAM/AS system versus CaCl_2_ concentration.

PAM and AS were added into aqueous CaCl_2_ solutions in different concentrations, and the time to loss of fluidity of the system was measured. Figure 3 shows the variation in the time to loss of fluidity of the system with CaCl_2_ concentration. As the CaCl_2_ concentration increases from 0.1 mol/L to 0.8 mol/L, the time to loss of fluidity of the system shows a trend of first rapidly shortening, significant prolongation, and then finally shortening again. Meanwhile, the microstructure and gelation mechanism of the gel undergo staged changes, which can be divided into three regions.

Low-Concentration Region (0.1 mol/L–0.3 mol/L): Within this range, the time to loss of fluidity is extremely short (<10 min), and a uniform, dense and continuous gel network forms rapidly (corresponding to the upper-left micrograph). This is because low-concentration Ca^2+^ can effectively promote the hydrolysis of the AS and accelerate the formation of polynuclear hydroxyaluminum complexes. These polynuclear complexes, as efficient crosslinking junctions, quickly undergo coordination reactions with functional groups such as carboxyl groups on PAM chains, constructing a three-dimensional network structure. As a result, the system achieves irreversible gelation within a short period.

Medium-Concentration Region (0.3 mol/L–0.6 mol/L): When the CaCl_2_ concentration exceeds 0.3 mol/L, the time to loss of fluidity is significantly prolonged (from approximately 5 min to about 120 min). Instead of forming a continuous gel, the system only shows water absorption and swelling of PAM particles without obvious crosslinking between particles (corresponding to the middle micrograph). This phenomenon arises because excessive Ca^2+^ inhibits the formation of polynuclear hydroxyaluminum complexes, leading to a sharp decline in the crosslinking activity of the aluminum crosslinker. At the same time, CaCl_2_ compresses the electric double layer of PAM, weakens the electrostatic repulsion between molecular chains, and causes chain-curling. Since the Ca^2+^ concentration has not yet reached the threshold for acting as an effective crosslinker, it cannot replace the role of the aluminum crosslinker. Consequently, stable three-dimensional networks cannot be formed between PAM chains, and the system only exhibits physical swelling of PAM itself.

High-Concentration Region (>0.6 mol/L, Blue Background Zone): When the CaCl_2_ concentration exceeds 0.6 mol/L, the time to loss of fluidity shortens again, and a crosslinked structure is re-established (corresponding to the lower-right micrograph). This is because high-concentration Ca^2+^ can act as a crosslinker [28]. However, the crosslinking bonds formed between Ca^2+^ and PAM are ionic bonds with weak intermolecular forces, belonging to physical crosslinking, resulting in relatively low overall gel strength.

### 2.2. Synthesis and Microstructure of the CaCl_2_-Tolerant Delayed-Release Crosslinker

#### 2.2.1. Adsorptive Loading of Crosslinker on Zinc Oxide

**Figure 4:**
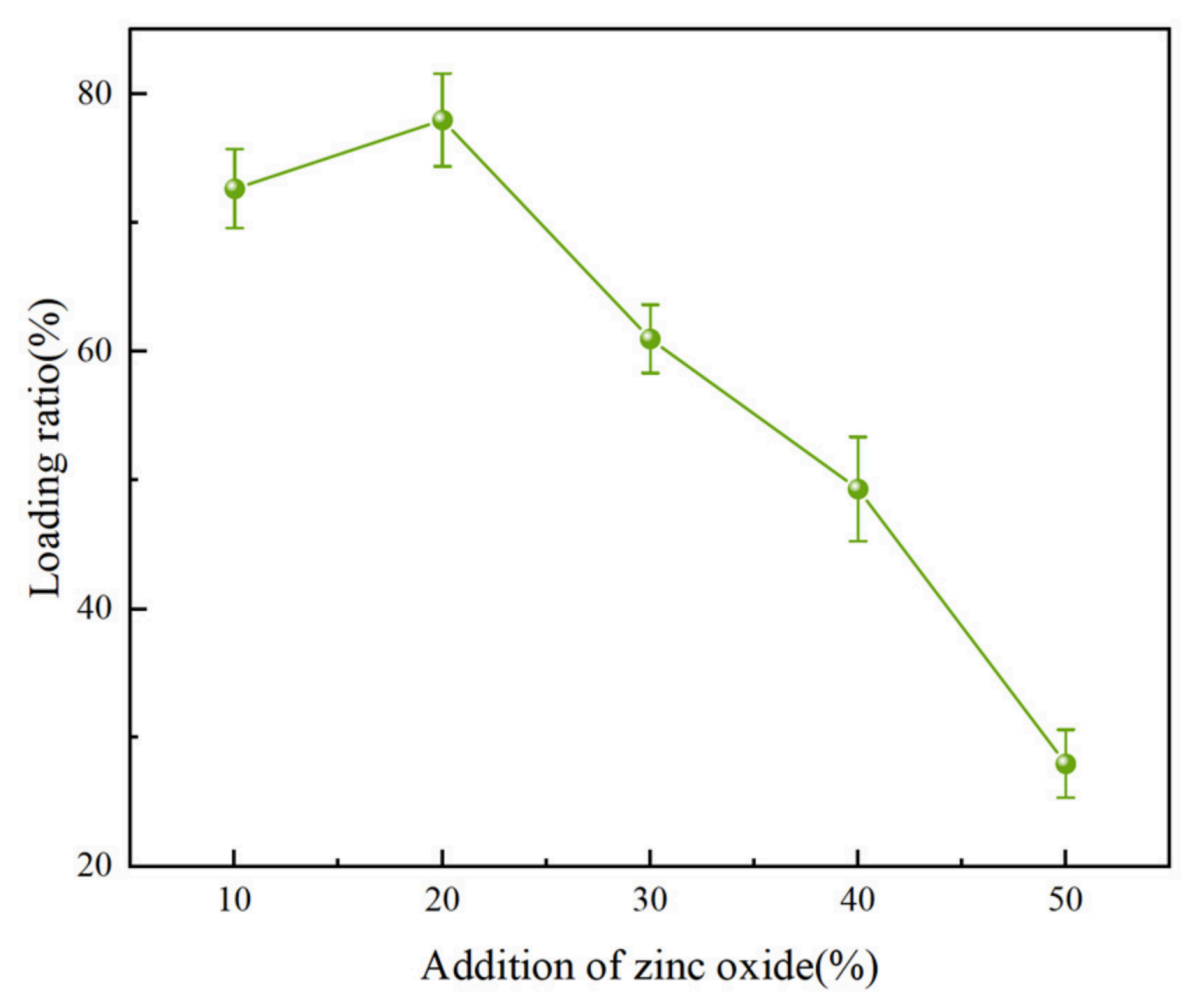
Variation in crosslinker loading ratio with zinc oxide addition.

As shown in Figure 4, as an adsorptive carrier for AS, zinc oxide (ZnO) provides active adsorption sites such as surface hydroxyl groups and structural defects. With an increase in ZnO dosage, the number of these sites rises, enabling more AS molecules to be bound, which results in a slight elevation of the loading ratio and a peak loading ratio of 72% at 20% ZnO addition. Since the AS solution is saturated, the total amount of adsorbable Al^3+^ in the system is fixed. When the ZnO dosage exceeds 20%, the total carrier content surpasses the maximum capacity of loadable AS, diluting the amount of AS allocated per unit mass of ZnO and directly leading to a decline in the loading ratio. Meanwhile, at high ZnO dosages, the particles are prone to agglomeration: small particles aggregate into larger ones, causing a substantial reduction in the effective specific surface area, a sharp decrease in the number of accessible active adsorption sites, and a significant drop in adsorption efficiency.

**Figure 5:**
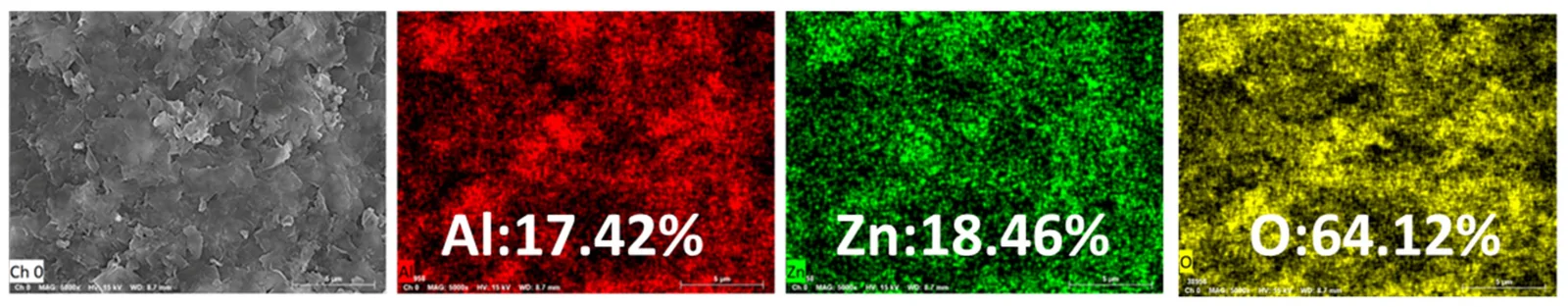
The surface morphology and elemental distribution of the AS supported on zinc oxide.

**Figure 6:**
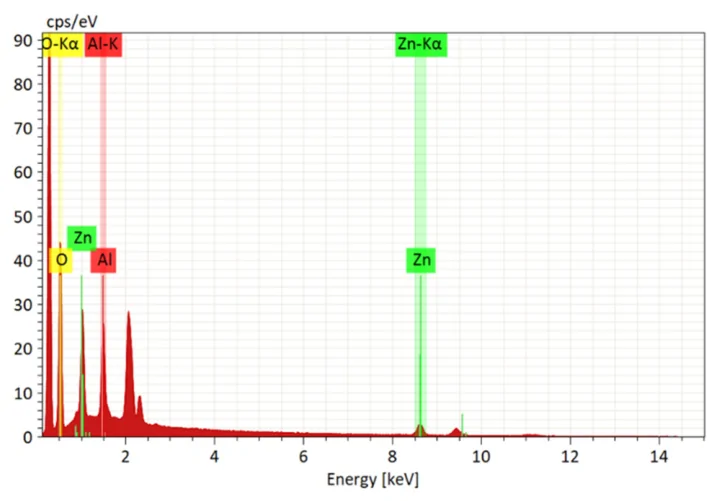
SEM-EDS spectrum of the AS supported on zinc oxide.

Figure 5 presents the surface morphology, elemental distribution, and corresponding EDS spectrum of AS-loaded ZnO. SEM observations combined with elemental mapping confirm the homogeneous dispersion of aluminum species on the ZnO surface, verifying efficient loading of the crosslinker. The EDS spectrum of Figure 6 further corroborates the presence of Zn, O, and Al elements with no obvious impurity signals: The significantly elevated oxygen signal in the EDS spectrum primarily stems from the formation of a polyhydroxy aluminum structure on the ZnO surface during the interaction with the aluminum crosslinker. This oxygen-rich functional group substantially boosts the local oxygen intensity. Meanwhile, the near 1:1 ratio of the zinc-to-aluminum peak intensities not only suggests the successful loading of aluminum species onto the ZnO surface but, more importantly, indicates that the two components are not merely physically mixed. Instead, it points to a dehydration reaction at the interface, leading to the formation of a chemically bonded Zn-O-Al bridging structure, which achieves molecular-level integration between the zinc oxide and the aluminum crosslinker.

**Figure 7:**
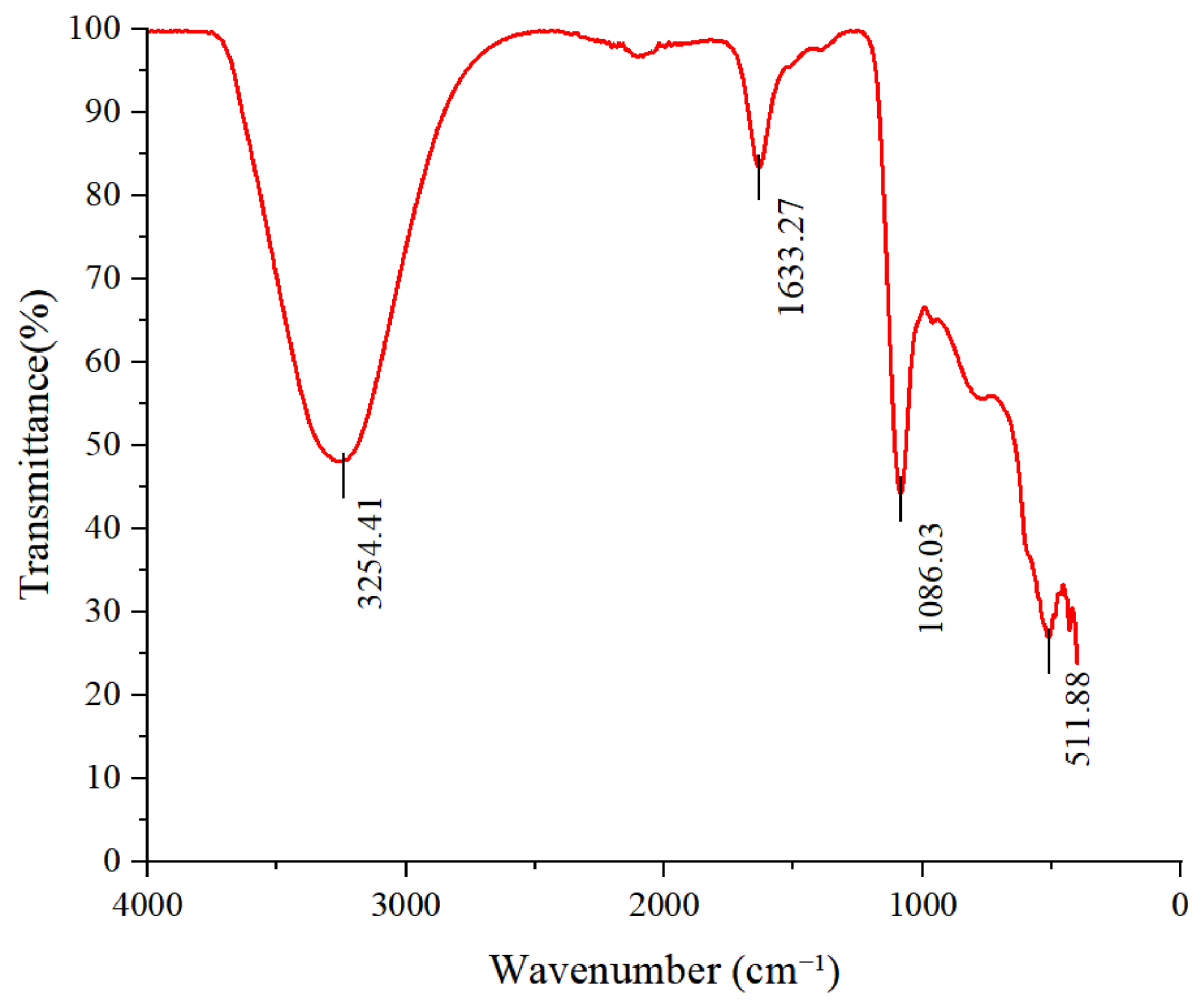
The FT-IR spectrum of the AS supported on zinc oxide.

Figure 7 shows the FT-IR spectrum of the AS supported on zinc oxide. The broad and strong characteristic absorption peak appearing at 3254.41 cm^−1^ [31] is mainly attributed to the O–H stretching vibration of adsorbed water and hydroxyl groups (–OH) on the sample surface. The broadening of this peak indicates the existence of intermolecular hydrogen bonding, reflecting the strong polarity and hydroxylation property of the zinc oxide surface. The absorption peak at 1633.27 cm^−1^ corresponds to the H–O–H bending vibration, further confirming the presence of adsorbed water on the sample surface, which corroborates the O–H stretching vibration peak at 3254.41 cm^−1^. The strong and sharp absorption peak observed at 1086.03 cm^−1^ is mainly ascribed to the stretching vibration of Al–O bonds or Al–O–Zn-bridging oxygen bonds. This characteristic peak demonstrates that chemical adsorption and interfacial bonding occur between the aluminum crosslinker and the zinc oxide surface, rather than simple physical adsorption. Aluminum species bind with hydroxyl groups on the zinc oxide surface via oxygen bridges to form a stable composite structure [32]. The absorption peak in the low-frequency region at 511.88 cm^−1^ belongs to the lattice vibration of metal–oxygen (M–O) bonds, mainly corresponding to the stretching vibration of Zn–O bonds in the zinc oxide matrix, and also involves the characteristic vibration of Al–O bonds in the supported aluminum species. This further verifies the successful loading and immobilization of the aluminum crosslinker on the zinc oxide surface.

**Figure 8:**
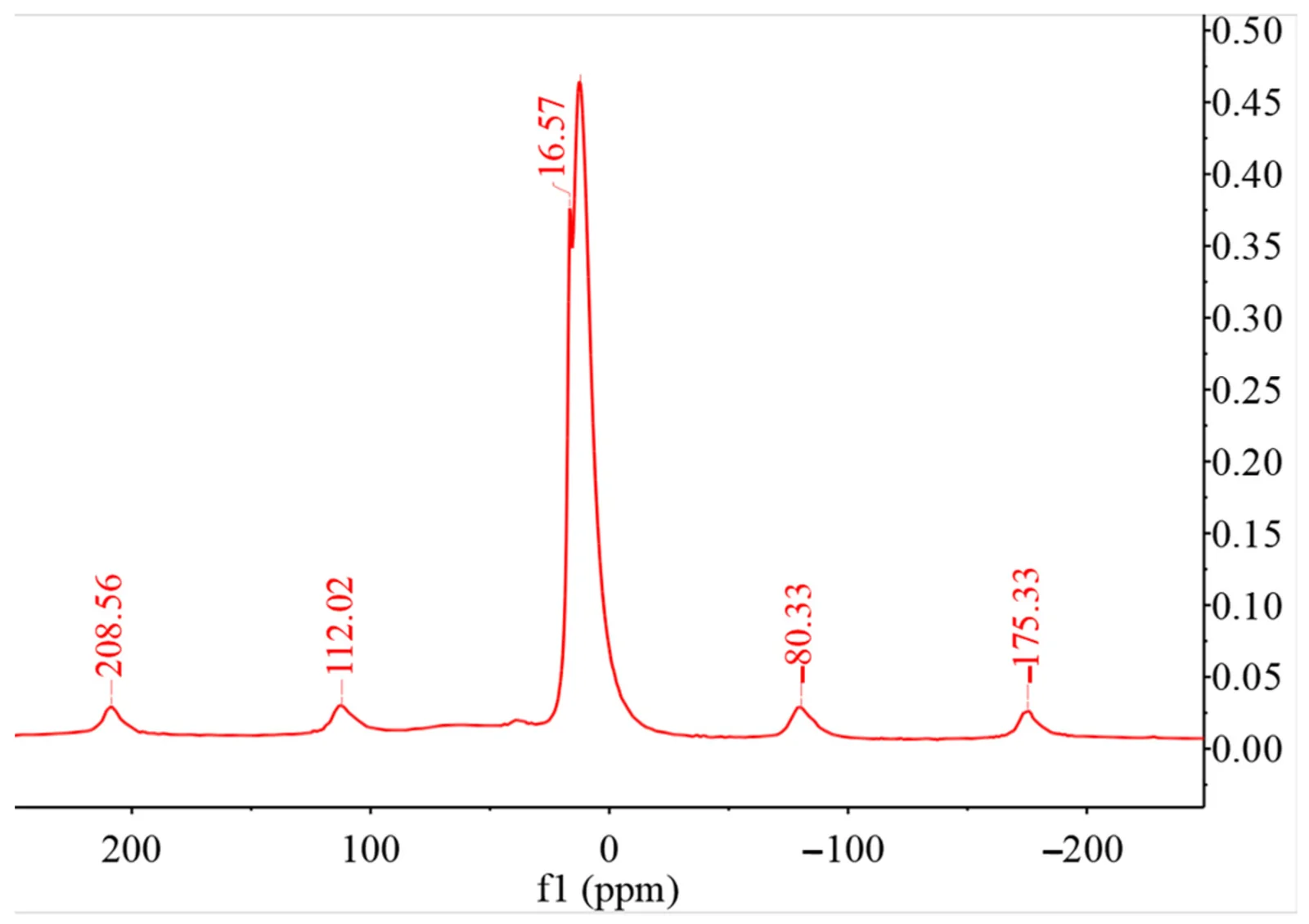
The solid-state ^27^Al MAS NMR spectrum of the AS adsorbed on zinc oxide.

The solid-state ^27^Al MAS NMR spectrum of the AS adsorbed on zinc oxide is shown in Figure 8. A sharp and strong peak at 12–16 ppm serves as the dominant characteristic signal, corresponding to octahedrally coordinated aluminum (AlO_6_) species. This chemical shift is slightly downfield relative to that of free hydrated Al^3+^ (typically near 0 ppm), suggesting a degree of coordinative interaction between the aluminum species and the ZnO surface, rather than simple physical adsorption. Notably, the characteristic tetrahedral aluminum (AlO_4_) resonance at +60 to +63 ppm, which is diagnostic of the Al_13_ Keggin-type polycation structure [33], was not observed in the spectrum, indicating that the aluminum species supported on the ZnO surface are not present as intact Al_13_-type Keggin clusters. The four weak, evenly spaced peaks at approximately ±100–210 ppm (marked with asterisks in Figure 8) correspond to spinning sidebands arising from magic-angle spinning and do not represent distinct aluminum chemical environments. The sharp peak shape and high intensity of the AlO_6_ signal indicate that the aluminum species adsorbed on the ZnO surface are dominated by octahedrally coordinated structures with a relatively symmetric local environment, weak quadrupolar interactions, and reasonable structural homogeneity, consistent with dispersed mononuclear or oligomeric hydroxyaluminum species rather than large Keggin-type polycations.

The ^27^Al MAS NMR spectrum directly verifies that ZnO possesses a stable loading capacity for polynuclear hydroxyaluminum species via a synergistic mechanism of hydrolytic prepolymerization, electrostatic adsorption and hydroxyl condensation:

(1) Al^3+^ coordinates with water molecules to form hydrated aluminum ions [Al(H_2_O)_6_]^3+^, which undergo stepwise deprotonation at room temperature under neutral to weakly alkaline conditions (matching the pH of the ZnO dispersion system) to generate mononuclear hydroxyaluminum ions [Al(H_2_O)_5_(OH)]^2+^ and [Al(H_2_O)_4_(OH)_2_]^+^. These mononuclear species may further polymerize via hydroxyl bridges to form hydroxyaluminum oligomers or polycations, such as the well-known Al_13_ polycation [Al_13_O_4_(OH)_24_(H_2_O)_12_]^7+^, which consists of one central tetrahedral Al and twelve peripheral octahedral Al units [33]. However, the absence of the AlO_4_ resonance in the present spectrum suggests that, under the adsorption conditions employed here, the hydrolyzed aluminum species interacting with the ZnO surface do not retain this fully condensed Keggin architecture. This is likely attributable to ligand exchange or surface complexation between the hydrolyzed aluminum species and surface Zn–OH groups during adsorption, which may disrupt the highly symmetric central AlO_4_ environment required to stabilize the Keggin structure, resulting instead in dispersed mononuclear or low-oligomeric octahedrally coordinated (AlO_6_) hydroxyaluminum species anchored on the ZnO surface. These AlO_6_-dominated species, rather than intact Al_13_ clusters, are proposed to serve as the core aluminum components contributing to the interfacial crosslinking network.

(2) The ZnO surface undergoes hydroxylation and dissociation in aqueous solution, forming negatively charged sites, which provides the basis for adsorbing the positively charged aluminum species. When ZnO is dispersed in water, surface Zn^2+^ binds with water molecules to form Zn–OH surface hydroxyl groups, and the dissociation of these hydroxyl groups renders the ZnO surface negatively charged. The hydroxyaluminum species formed by hydrolysis of the AS have positive charges on their surfaces. Based on the principle of electrostatic attraction between opposite charges, they migrate and adsorb onto the ZnO surface, achieving preliminary loading and enrichment of aluminum species and preventing their agglomeration and loss. This adsorption is selective, favoring positively charged aluminum species, which is consistent with the absence of extraneous aluminum signals in the spectrum.

(3) Electrostatic adsorption only achieves preliminary loading; the key to stable loading lies in the hydroxyl condensation crosslinking reaction, which forms covalent bonds between aluminum species and the ZnO surface, thereby immobilizing the aluminum species. The aluminum polycations adsorbed on the ZnO surface contain abundant hydroxyl groups, which are highly reactive with the undissociated Zn–OH groups on the ZnO surface and undergo dehydration condensation at room temperature:

(4) Zn–OH + HO–Al⇌Zn–O–Al + H_2_O

The reaction presented in (4) is essentially a nucleophilic substitution: the oxygen atom in Zn–OH (with lone pair electrons) attacks the empty orbital (sp^3^ hybridized) of the Al atom, forming a Zn–O–Al covalent bond and realizing the chemical immobilization of the aluminum crosslinker.

#### 2.2.2. Delayed Release of Crosslinker

As the system temperature rises, the Zn–O–Al covalent bonds on the ZnO surface begin to break, and hydroxyaluminum desorbs from the ZnO surface and is gradually released into the solution system. The hydroxyaluminum released into the system retains its reactivity and diffuses gradually in the solution. When its concentration reaches a certain level, the hydroxyl groups on its surface undergo coordination crosslinking reactions with the amide groups and carboxyl groups on PAM molecular chains, eventually forming a three-dimensional network gel structure. This prevents premature gelation of the system caused by the rapid release of the crosslinker and achieves delayed crosslinking of the gel plugging system.

#### 2.2.3. Delayer Effect of CaCl_2_ Concentration on Delayed-Release Kinetics

Unlike the free AS system described in Section 2.1, where Al^3+^ hydrolysis and network formation occur directly within the CaCl_2_-containing environment and are therefore governed by Ca^2+^ concentration, the hydroxyaluminum species in the ZnO-supported system are pre-formed and covalently immobilized onto the ZnO surface via Zn–O–Al bonds (Section 2.2.1) prior to introduction into the CaCl_2_-containing system. Because this hydrolysis and immobilization step occurs independently of the final system’s Ca^2+^ concentration, the resulting Al species are not subject to the same Ca^2+^-induced inhibition observed in the free-AS system.

Within this ZnO-supported system, CaCl_2_ instead plays a role in modulating the delayed-release kinetics of Al species from the ZnO carrier, and correspondingly the delayed gelation time of the system. Among the CaCl_2_ concentrations evaluated, 6 mol/L was identified as optimal—not because it coincides with a favorable network-formation window, as classified in Section 2.1.2, but because it produced the longest delayed gelation time, which is advantageous for field applications as it allows sufficient placement time prior to gelation.

#### 2.2.4. Cryo-SEM Structure of the Gel

After the gel samples were crosslinked, their microstructure was characterized using a cryo-scanning electron microscope (Cryo-SEM, Model SU3500, Hitachi High‑Technologies Corporation, Tokyo, Japan). The gel samples were first mounted onto a specialized cryo-stage holder and rapidly plunged into liquid nitrogen slush at −196 °C for vitrification rapid freezing so as to avoid ice crystal formation and maintain the original microstructure. Under the protection of liquid nitrogen, the frozen samples were transferred into a cryo-preparation chamber. After removing the surface-adsorbed ice layer by low-temperature sublimation, the samples were sputter-coated with a thin layer of platinum to improve electrical conductivity. Subsequently, the samples were transferred into the microscope chamber equipped with a cryo-stage, and the crosslinked network structure of the gel was observed under constant low-temperature conditions.

**Figure 9:**
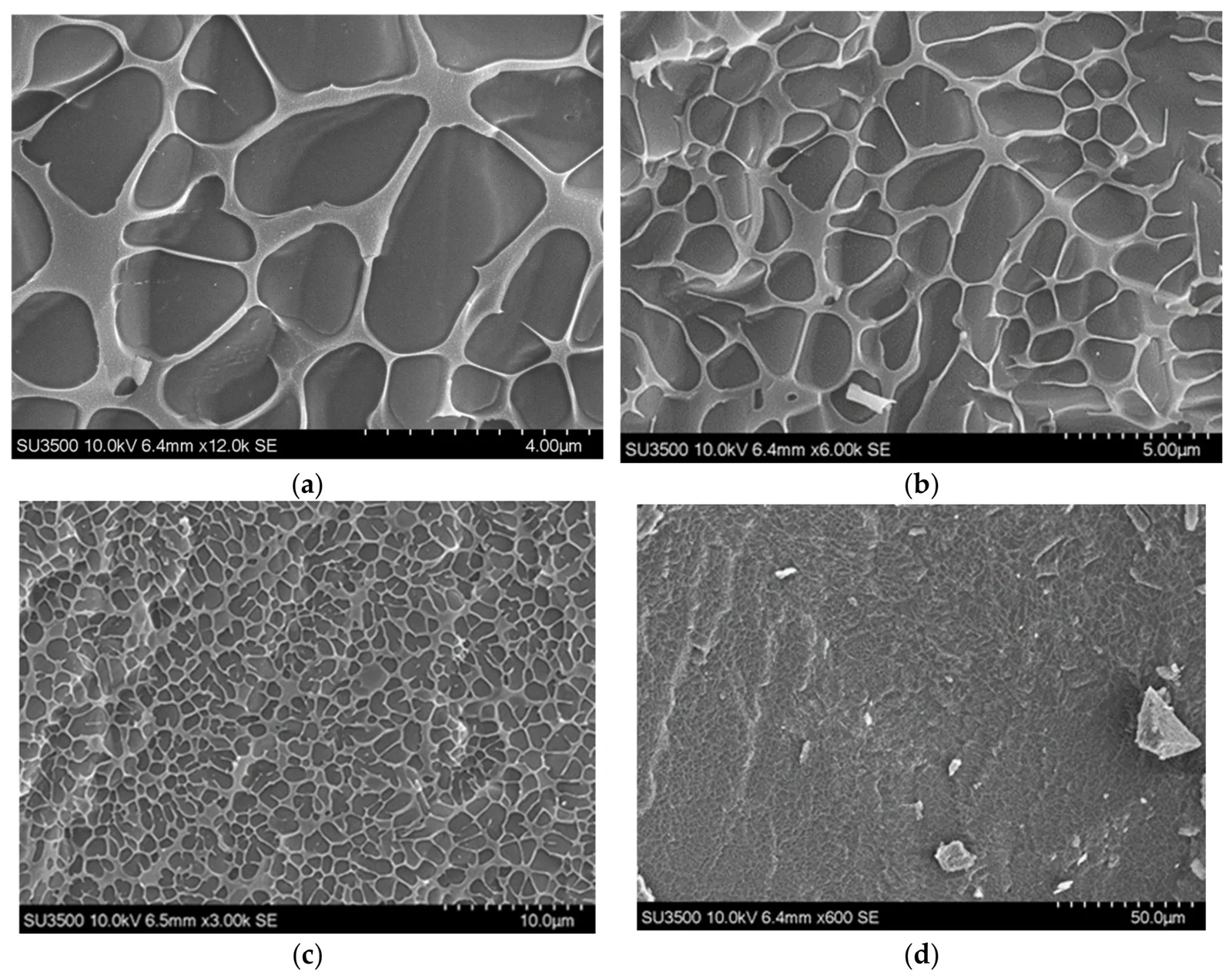
Cryo-SEM image. (**a**) Large-pore network structure at ×12.0k magnification; (**b**) medium-pore network structure at ×6.0k magnification; (**c**) dense small-pore network structure at ×3.0k magnification; (**d**) overall compact gel morphology at ×600 magnification.

The cryo-SEM image in Figure 9 exhibits a typical honeycomb-like three-dimensional interconnected porous network structure, which is the pore morphology formed by the sublimation of ice crystals after freeze-drying the hydrogel, fully retaining the original crosslinked skeleton of the gel. The pore walls are composed of a continuous PAM–aluminum crosslinker composite skeleton, interwoven to form a dense three-dimensional network without obvious skeleton fracture or pore collapse, reflecting a high degree of crosslinking and good structural stability.

In terms of pore structure and size, the equivalent diameter of a single pore is approximately 1–5 μm, belonging to a micron-scale porous structure. The pore shapes are mostly irregular ellipses or polygons without regular circular pores, consistent with the natural morphology of ice crystal growth during freeze-drying. The pore walls are thin and continuous, with high connectivity between skeletons, forming a through pore network that not only ensures the mechanical support of the gel but also provides channels for ion and fluid transport. Meanwhile, the pores and skeletons are uniformly distributed overall, with no obvious macropore agglomeration or local sparse regions, indicating uniform kinetics of the crosslinking reaction and synchronous stability of phase separation and network formation during gelation.

### 2.3. Rheological Characteristics and Gelation Time Evaluation of Delayed Crosslinking System

#### 2.3.1. Rheological Characteristics

**Figure 10:**
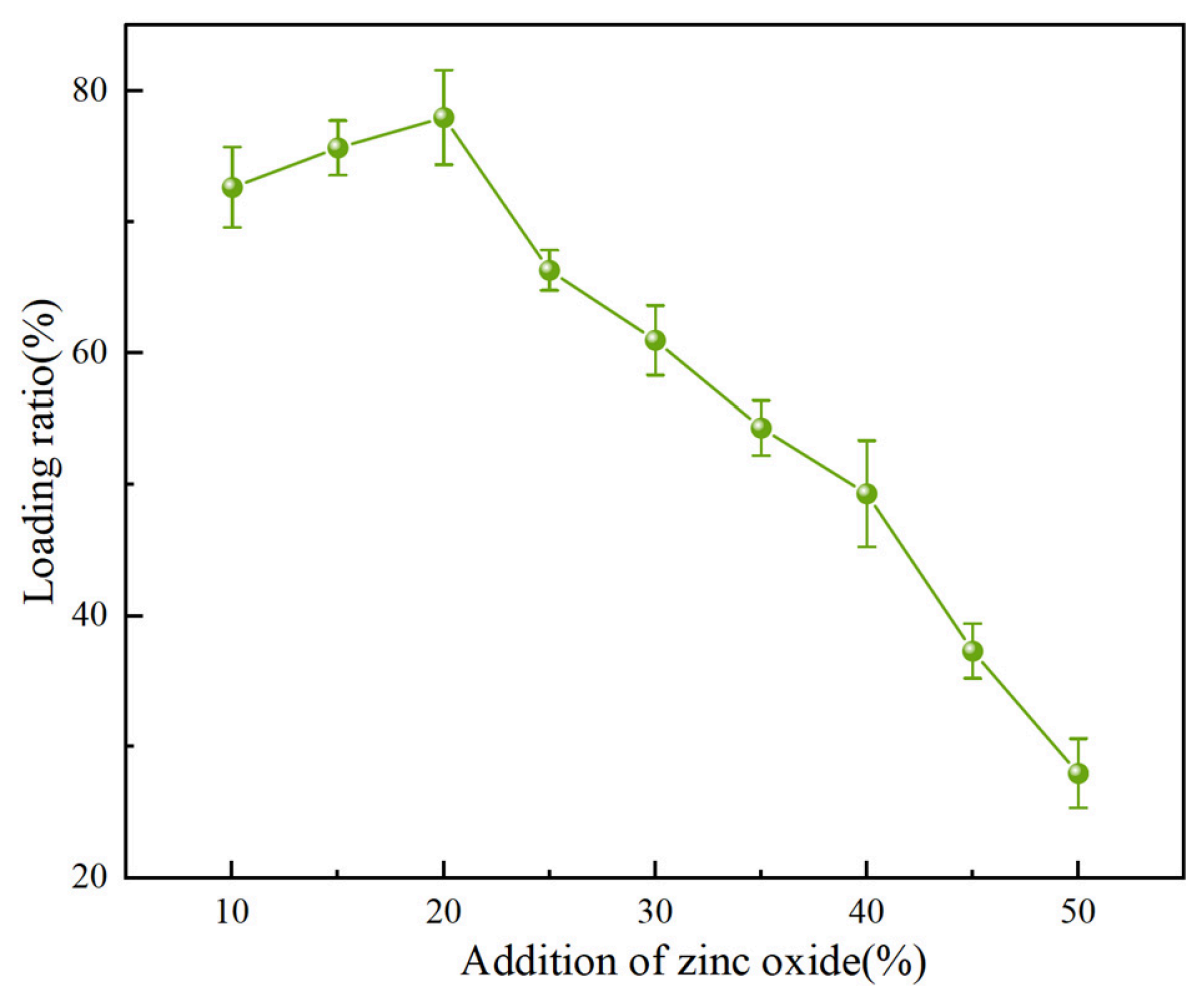
Viscosity evolution of the delayed crosslinking system at different temperatures.

Figure 10 shows that, at 30 °C, the viscosity remained stable at approximately 20 mPa·s without significant increase, indicating that the crosslinker was effectively immobilized and barely released at ambient temperature, ensuring long-term pumpability for wellbore delivery. At 60 °C, the viscosity increased gradually with time, showing a mild and progressive thickening behavior, which suggests the slow release of the crosslinker and the gentle initiation of crosslinking reaction at moderate temperature. When the temperature rose to 90 °C, the viscosity increased significantly, from 29 mPa·s to 49 mPa·s within 100 min, with a much higher growth rate than that at lower temperatures, reflecting the acceleration effect of high temperature on crosslinker release and crosslinking reaction. These results demonstrate that the system exhibits obvious temperature-responsive viscosity evolution, which can maintain low viscosity for safe pumping in the wellbore and gradually thicken in high-temperature formations.

#### 2.3.2. Flow Loss Time and Gelation Time

**Figure 11:**
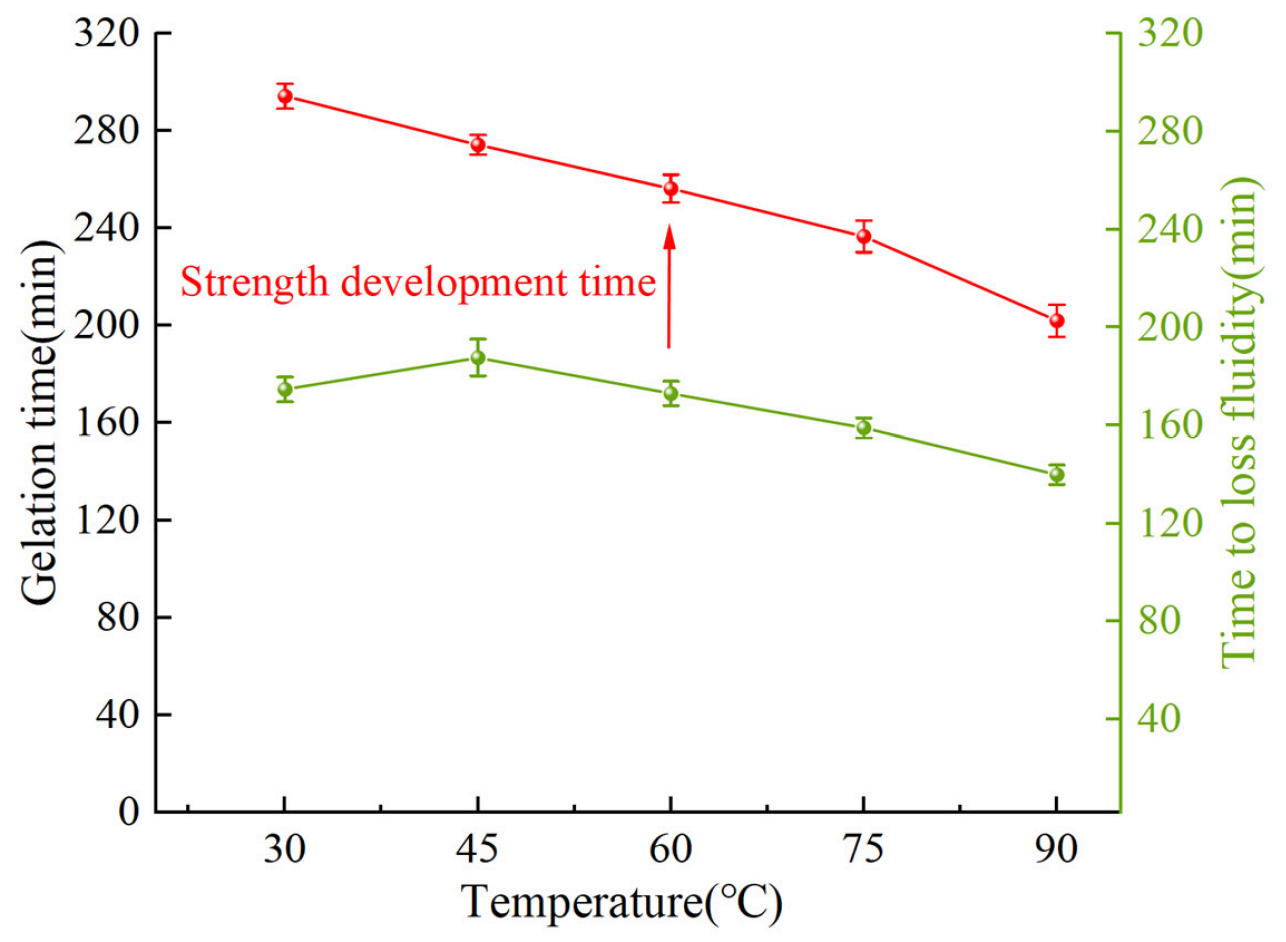
Variations in time to loss of fluidity and gelation time of the delayed crosslinking system with temperature.

Figure 11 shows the variations in time to loss of fluidity and gelation time of the delayed crosslinking system with temperature. The gelation behavior of the system is temperature-dependent. With increasing temperature, both the gelation time and the time to loss of fluidity are shortened, and the development of gel strength is accelerated.

The rise in temperature enhances molecular thermal motion. Although the PAM molecular chains remain coiled, their water absorption and viscosity increase behavior is intensified, resulting in a shorter time to loss of fluidity. Meanwhile, the release rate of the aluminum crosslinker from zinc oxide is accelerated, the activation energy of the crosslinking reaction is lowered, and the three dimensional gel network grows at an accelerated rate, thereby shortening the development time of gel strength.

### 2.4. Evaluation of Performance of the Gel

#### 2.4.1. Evaluation of Temperature Resistance of the Gel

**Figure 12:**
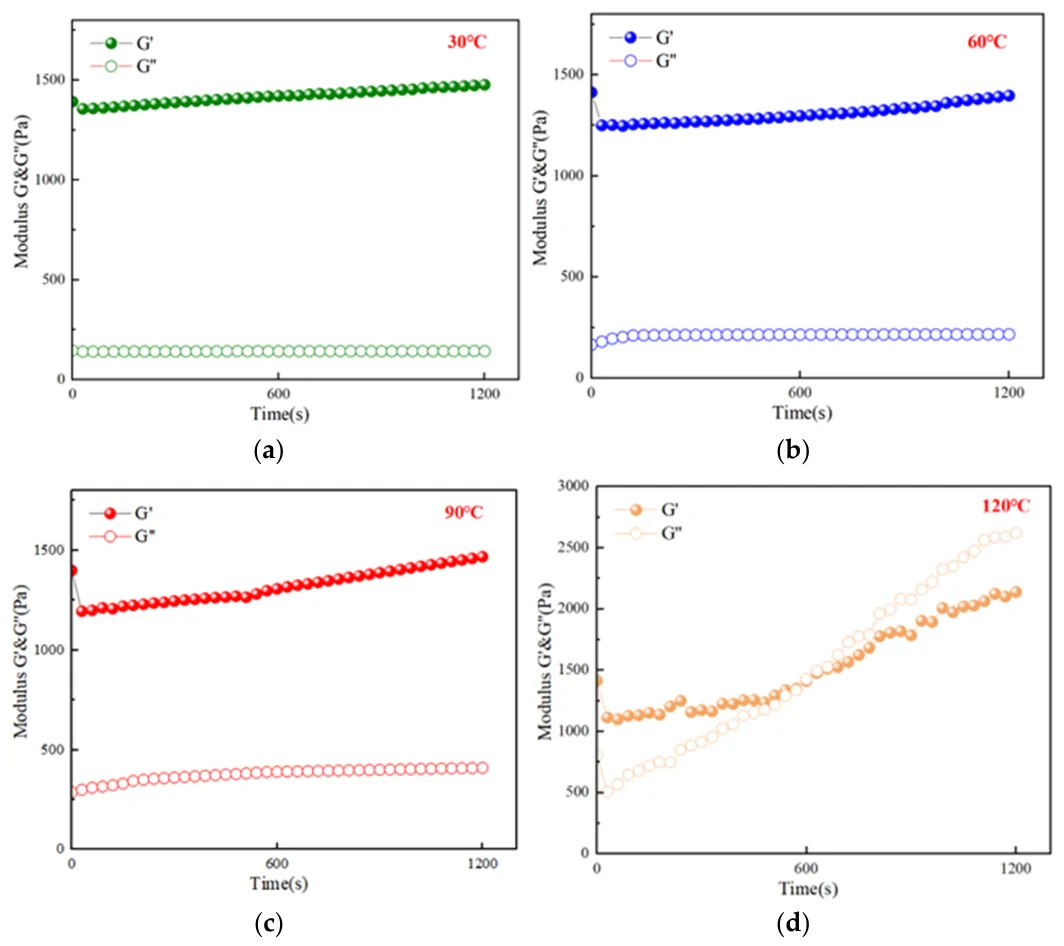
Modulus variation in gels at different temperatures.

As shown in Figure 12a,b, at low temperatures of 30 °C and 60 °C, the gel remains relatively stable. G′ is steadily maintained at 1300–1500 Pa and shows only a slow slight increase with testing time, while G″ stays at a low level of 100–200 Pa. G′ is much larger than G″, and the modulus curves are generally gentle. This behavior indicates that at 30–60 °C, the three-dimensional crosslinking network formed by Al^3+^ coordination bonds is highly stable, with crosslinking sites resistant to breakage or rearrangement, and the system exhibits typical rigid gel characteristics. When the temperature rises to 90 °C (Figure 12c), the growth rate of G′ with time increases significantly, and G″ also rises to a certain extent, slowly increasing from about 300 Pa to approximately 400 Pa. However, G′ is still notably higher than G″, and the system remains in an elasticity-dominated gel state. This shows that when the temperature rises to a certain level, the three-dimensional crosslinking network formed between PAM and Al^3+^ becomes denser and more stable. Meanwhile, the intensified thermal motion of molecular chains leads to a certain increase in G″.

Under the high-temperature condition of 120 °C (Figure 12d), G′ rises from about 1200 Pa to approximately 2200 Pa with obvious fluctuations. Although G″ is lower than G′ at the initial stage, its growth amplitude is far greater than that of G′, and it surpasses G′ at around 600 s. The gel finally shows a non-gelling state. This rheological behavior is consistent with a mechanism in which the Al^3+^ coordination bonds undergo thermal aging at high temperatures, with partial crosslinking bonds hydrolyzed and broken, thereby limiting the growth of G′—the elastic supporting capacity of the three-dimensional network. In addition, the intensified thermal motion of molecular chains greatly enhances segmental relaxation and interchain friction, causing a sharp rise in the viscous modulus G″. The system eventually presents a non-gelling state dominated by the viscous modulus.

**Figure 13:**
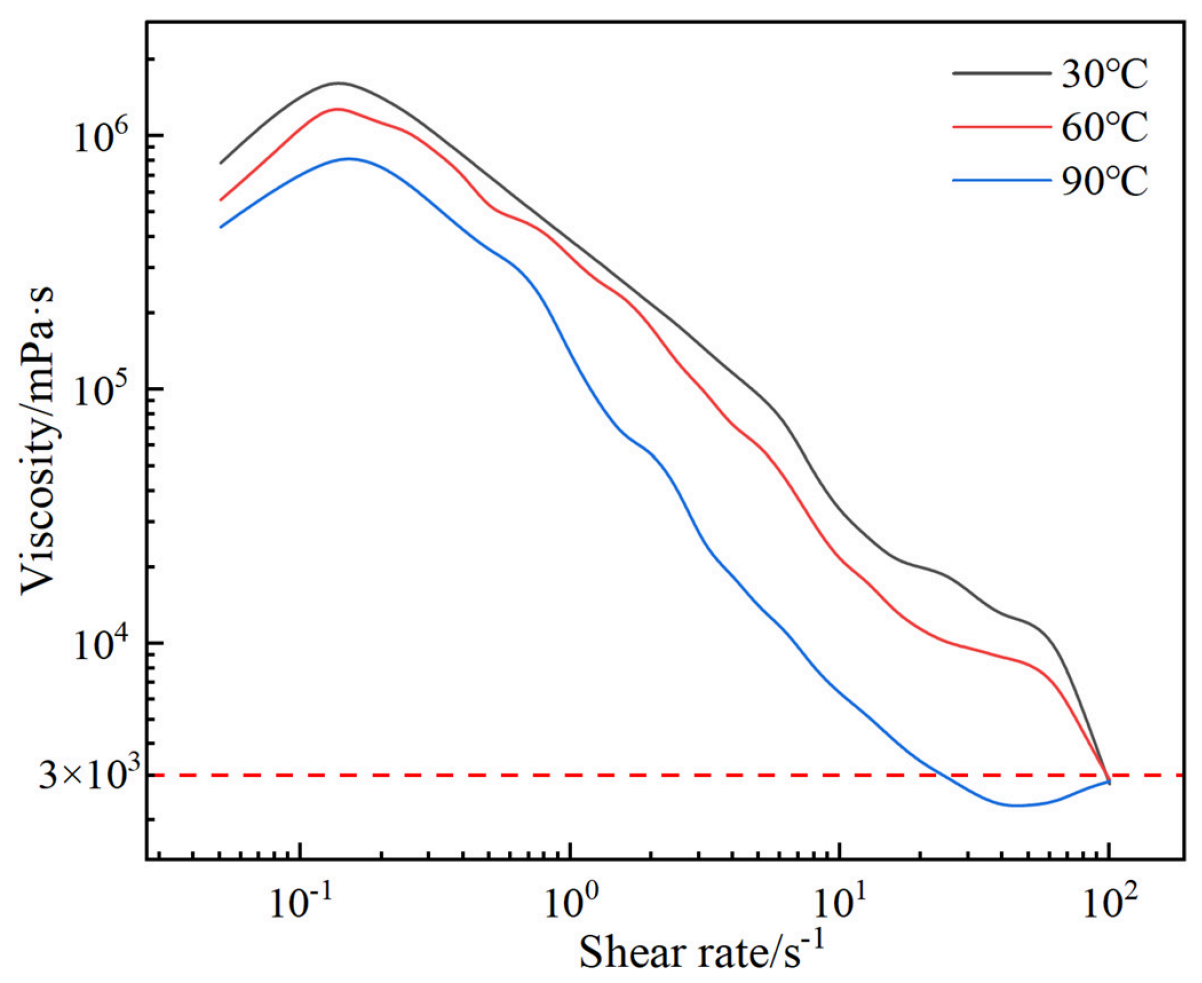
Steady shear rheological curves of gels at different temperatures.

The steady shear rheological behavior of the gels, measured at 30, 60, and 90 °C, is presented in Figure 13, with apparent viscosity plotted as a function of shear rate over the range of 0.1–100 s^−1^. All three profiles exhibit typical shear-thinning (pseudoplastic) behavior, a key property for profile control and water shutoff applications. At low shear rates (<0.2 s^−1^), a slight initial increase in viscosity is observed, which is attributed to the gradual activation and transient reinforcement of the crosslinked network, as polymer chains begin to align and entangle under weak shear forces. Beyond this low-shear regime, the apparent viscosity decreases monotonically with increasing shear rate, reflecting the progressive disruption of the three-dimensional crosslinked structure, the disentanglement of polymer chains, and their orientation along the flow direction. Temperature exerts a significant influence on the rheological performance: at any given shear rate, the apparent viscosity follows the order 30 °C > 60 °C > 90 °C, as elevated temperatures intensify molecular thermal motion, weakening intermolecular hydrogen bonding, physical entanglements, and weak crosslinking interactions, thereby reducing flow resistance. Critically, the gel demonstrates robust shear resistance across the tested temperature range. At 30 °C, the gel maintains the highest apparent viscosity throughout the shear rate sweep, with the slowest viscosity decay rate, and retains a viscosity above 3 × 10^3^ mPa·s even at 100 s^−1^, indicating excellent structural stability. While the overall viscosity decreases and the decay rate accelerates with increasing temperature to 60 and 90 °C, the gel still maintains a viscosity of approximately 3 × 10^3^ mPa·s at the highest shear rate, without catastrophic structural failure. This favorable shear resistance and viscosity retention under both moderate and high shear conditions confirm that the aluminum-crosslinked gel can withstand the mechanical forces encountered during pumping and reservoir propagation, making it well-suited for use in enhanced oil recovery operations.

#### 2.4.2. Evaluation of Plugging Strength of the Gel

The evaluation results in Table 1 showing gel plugging performance under different temperatures and quartz sand mesh sizes show that the gel system exhibits excellent plugging effect in the range of 30–90 °C, with a plugging rate higher than 90% and the breakthrough pressure reaching more than 5 MPa. Within the range of 30–60 °C, the gel crosslinking reaction gradually becomes sufficient with increasing temperature, and the three-dimensional network structure is densified, which manifest through an increase in breakthrough pressure, decrease in permeability after breakthrough, and plugging rate stably ranging from 93% to 95.6%. However, when the temperature rises to 90 °C, the high temperature accelerates the thermal degradation of HPAM molecular chains and the hydrolysis of amide groups, weakening the mechanical support effect of the polymer skeleton and leading to a significant decrease in elastic modulus (G′), which ultimately manifests as a decrease in breakthrough pressure and a slight reduction in plugging efficiency. Notably, no G′–G″ crossover is observed at 90 °C, distinguishing this backbone-degradation-dominated process from the crosslink-decoupling behavior identified at 120 °C.

These results collectively confirm that, although the gel lacks the rigid consolidated strength characteristic of cement-like materials, its elastic structural strength—governed primarily by G′—is sufficient to maintain effective sealing performance across a wide temperature range, consistent with the breakthrough pressure and plugging efficiency data presented above. It should be noted that the plugging evaluation in this study was conducted using quartz-sand packs rather than fractured cores or fracture-slot models, with simulated formation water only (no drilling-fluid contamination) and a single static aging window (4–8 h). While this system allows for a controlled and reproducible assessment of the delayed-crosslinking gelation mechanism and its dependence on pore/fracture-equivalent opening size, it does not capture the surface roughness, connectivity, and heterogeneity of real fractures, nor the potential interference of drilling-fluid components, nor the sensitivity of gelation and plugging performance to aging time. Fractured-core or fracture-slot displacement experiments, testing under drilling-fluid-contaminated conditions, and an aging-time sensitivity study are therefore identified as necessary next steps before the system’s field-scale plugging performance in fractured reservoirs can be fully established.

#### 2.4.3. Comparative Evaluation Against Established Delayed-Crosslinking Systems

To benchmark the plugging performance of the ZnO-supported delayed-crosslinking system against established approaches, we conducted a side-by-side comparison under identical experimental conditions (HPAM: 2000 mg/L, temperature: 70 °C, salinity: 5000 mg/L NaCl). Four representative delayed-crosslinking systems were selected for comparison: (1) ligand-shielded organic zirconium (Zr^4+^) crosslinker; (2) ligand-shielded organic chromium (Cr^3+^) acetate crosslinker; (3) redox-regulated Cr^6+^/Cr^3+^ system; and (4) ethyl cellulose-coated microcapsule Cr^3+^ crosslinker. The comparative results are summarized in Table 2.

As shown in Table 2, the ZnO-supported system exhibits several distinctive advantages. First, the delay time (20–50 h) is broadly tunable and covers the typical operational window required for deep reservoir penetration, comparable to or exceeding that of microcapsule-coated systems (16–32 h), while avoiding the poor controllability of redox Cr^6+^/Cr^3+^ systems (2–48 h with large variance). Second, the ZnO-supported system maintains stable delayed crosslinking up to 120 °C, significantly outperforming ligand-shielded Cr^3+^ (≤80 °C) and redox systems (≤70 °C), and surpassing ligand-shielded Zr^4+^ (≤90 °C). Third, the breakthrough pressure of the ZnO-supported gel (2.8–3.5 MPa/m) is higher than that of ligand-shielded Zr^4+^ (1.8–2.5 MPa/m), microcapsule-coated Cr^3+^ (1.8–2.5 MPa/m), and redox systems (1.5–2.0 MPa/m), and is competitive with the highest reported Cr^3+^-based gels (up to 3.13 MPa/m). Fourth, from a cost perspective, the ZnO-supported system uses inexpensive ZnO as the carrier material, avoiding the high costs associated with organic ligand synthesis, microencapsulation processing, and the additional handling and disposal costs of toxic Cr^6+^ species. These comparative results collectively demonstrate that the ZnO-supported delayed-crosslinking system offers a superior comprehensive plugging performance—balancing tunable delay time, broad temperature tolerance, high plugging efficiency, and low material cost—that is not simultaneously achieved by any single existing approach.

## 3. Conclusions

1. High-concentration CaCl_2_ solution is used to carry high-molecular-weight PAM. While forming high-strength gel, it inhibits the hydrolytic viscosity increase in high-molecular-weight PAM, maintains the fluidity of the system, and ensures its smooth pumping into the wellbore and entry into the lost circulation zone. A CaCl_2_ concentration of less than 0.3 mol/L can promote the hydrolysis of Al^3+^ to form polynuclear hydroxyaluminum. At a CaCl_2_ concentration of 0.6 mol/L, the hydrolysis of Al^3+^ is inhibited, allowing the PAM/inorganic aluminum ion crosslinker system to maintain fluidity for approximately 300 min (Figure 3). When the CaCl_2_ concentration exceeds 0.6 mol/L, calcium ions can act as a new crosslinker, causing the system to lose fluidity rapidly and form a weakly crosslinked structure.
2. A CaCl_2_-tolerant slow-release crosslinker was synthesized. ZnO is employed to adsorb and carry the inorganic aluminum ion crosslinker in the form of octahedrally coordinated aluminum (AlO_6_) at 70 °C. In the saturated solution of the AS, the loading ratio of the crosslinker onto zinc oxide peaks at a ZnO addition of 20%, was 72%. This avoids the inhibition of hydrolysis of the AS induced by high-concentration CaCl_2_ solution, and a stable Zn–O–Al chemical bond is formed on the surface of zinc oxide, realizing the delayed release of the crosslinker.
3. In the temperature range of 30–90 °C, the delayed crosslinking system maintains favorable fluidity (measured by the visual code method), with the time to loss of fluidity longer than 120 min and the gelation time longer than 200 min. The elastic modulus G′ and viscous modulus G″ of the formed gel remain relatively stable, and the system exhibits excellent plugging performance with a plugging rate higher than 90% and a breakthrough pressure above 5 MPa. However, when the temperature reaches 120 °C, the viscous modulus of the gel exceeds the elastic modulus, and the system transforms into a non-gelling state.
4. Future work should include fractured-core or fracture-slot displacement testing under conditions more representative of field application (including drilling-fluid contamination and a range of aging times) to further validate the plugging performance and field applicability of the delayed-crosslinking gel system reported here.

## 4. Materials and Methods

### 4.1. Materials and Equipments

Polyacrylamide (PAM, 120 mesh, molecular weight = 1.7 × 10^7^ Da, hydrolysis degree = 25%); inorganic aluminum crosslinker (AS, The crosslinker was prepared by partial alkalization of aluminum sulfate (Al_2_(SO_4_)_3_·18H_2_O, analytical grade, purity ≥ 99%, yielding an alkalized polyaluminum hydroxide (PAH) species with the following characteristics: Al_2_O_3_ content of 16%, a basicity (B value) of 59%, and pH 4.3); calcium chloride (CaCl_2_, analytical grade, purity ≥ 99.5%) and nano zinc oxide (ZnO, analytical grade, purity ≥ 99.7%) were purchased from Yousuo Sample; deionized water (resistance ≥ 18.2 MΩ·cm) used in all experiments was prepared by a Milli-Q ultrapure water system.

Magnetic stirrer (Model MS‑H‑PROA, Beijing DLAB Scientific Co., Ltd., Beijing, China); HAAKE rheometer (Model HAAKE MARS 60, Thermo Fisher Scientific Inc., Karlsruhe, Germany); core flooding apparatus (Model HAD‑2, Hai’an Petroleum Scientific Research Instrument Co., Ltd., Hai’an, China); fractured core; chilled mirror scanning instrument (Model SDL‑100, Shanghai Precision & Scientific Instrument Co., Ltd., Shanghai, China); biological microscope (Model Olympus BX53, Olympus Corporation, Tokyo, Japan); UV‑Vis spectrophotometer (Model UV‑2600, Shimadzu Corporation, Kyoto, Japan); constant‑temperature water bath (Model HH‑S2, Changzhou Guoyu Instrument Manufacturing Co., Ltd., Changzhou, China); scanning electron microscope (SEM, Model SU8010, Hitachi High‑Technologies Corporation, Tokyo, Japan); Fourier transform infrared (FT‑IR) spectrometer (Model Nicolet iS50, Thermo Fisher Scientific Inc., Waltham, MA, USA); nuclear magnetic resonance (NMR) spectrometer (Model Bruker AVANCE III 400 MHz, Bruker Corporation, Billerica, MA, USA); cryo‑scanning electron microscope (Cryo‑SEM, Model SU3500, Hitachi High‑Technologies Corporation, Tokyo, Japan).

### 4.2. Optimization of CaCl_2_ Concentration

A total of 10.4%PAM and 1.3%AS were added into CaCl_2_ solutions at different concentrations, and the time required for the system to lose fluidity was measured. Experiments in each group were performed in triplicate. The gel after losing fluidity was observed under a microscope to examine its state and morphology.

### 4.3. Synthesis of the CaCl_2_-Tolerant Delayed-Release Crosslinker

The AS was fully dissolved in deionized water until saturation to obtain a saturated AS solution. Zinc oxide powder with a mass denoted as *m*_1_ was added into the saturated solution. The mixture was magnetically stirred at 70 °C for 2 h to enable sufficient hydrolysis of the AS into polynuclear hydroxyaluminum complexes, which were subsequently loaded onto the ZnO surface. The zinc oxide adsorbing and supporting the AS was collected by filtration, then dried in an oven at 80 °C. The mass of the loaded zinc oxide was recorded as *m*_2_. The loading ratio *η* was calculated by Equation (5). Experiments in each group were performed in triplicate.

(5) $$\eta =\frac{m_{2}-m_{1}}{m_{1}}\times 100\%$$

In this formula, η—loading ratio, %;

*m*_1_—mass of added zinc oxide, g;

$m_{2}$ —mass of zinc oxide after loading crosslinker, g.

### 4.4. Preparation of Delayed Crosslinking System

Amounts of 10.4% PAM and 3% CaCl_2_-tolerant delayed-release crosslinker were added into a 6 mol/L CaCl_2_ solution to obtain a delayed gelation system. In the formulation, the composite crosslinking agent was used at a dosage of 3%. Considering the maximum loading rate (72%) presented in Figure 4 and the practical losses during preparation, the actual aluminum crosslinker content was estimated to be ~2%, which is slightly above the optimal level (1.3%) identified in Section 4.2. This difference is attributed to the steric hindrance and diffusion constraints caused by the ZnO support, which lower the crosslinking efficiency per unit of aluminum. Hence, a higher total dosage is required to attain an equivalent crosslinking density. A time-sweep experiment was then performed at a constant shear rate of 170 s^−1^ to evaluate the initial viscosity of the solution prior to gelation. Experiments in each group were performed in triplicate.

It is important to distinguish the mechanistic role of CaCl_2_ in the two crosslinking systems examined in this study. In the free AS system (Section 2.1), Al^3+^ hydrolysis and subsequent network formation take place directly within the CaCl_2_-containing environment; Ca^2+^ concentration therefore directly governs the extent and pathway of Al hydrolysis, giving rise to the network-formation windows classified in Section 2.1.2. By contrast, in the ZnO-supported AS system (Section 2.2), hydroxyaluminum species are pre-formed and covalently immobilized onto the ZnO surface prior to introduction into the CaCl_2_-containing system. This decoupling of the hydrolysis/immobilization step from the final Ca^2+^ concentration renders the ZnO-supported Al species insensitive to the Ca^2+^-induced inhibition observed in the free system. Instead, CaCl_2_ in this system primarily modulates the delayed-release kinetics of Al species from the ZnO carrier, thereby controlling the delayed gelation time; the 6 mol/L CaCl_2_concentration was therefore selected on the basis of delayed-gelation performance rather than network-formation behavior. This distinction clarifies why the optimal CaCl_2_ concentrations differ between the two systems and reflects the different underlying mechanisms governing crosslinking control in each case.

### 4.5. Determination of the Time to Loss of Fluidity of the Delayed Crosslinking System

The time to loss of fluidity of the gel was determined by the visual code method, as shown in Table 3 [32]. This time is a core performance index for the system to transform from a flowable liquid (Code A) to an almost non-flowable gel (Code E), which directly determines whether the system can be smoothly pumped downhole and effectively extend plugging in the lost circulation zone. A sufficiently long time to loss of fluidity can ensure that the system maintains a low-viscosity flowing state throughout the entire process of surface preparation and wellbore pumping, avoiding excessively high pump pressure, pipeline blockage or premature plugging of the near-wellbore zone caused by premature crosslinking and gelation, and ensuring that the system can stably migrate to the deep lost circulation zone. If the time to loss of fluidity is too short, it is prone to premature gelation leading to injection failure; if the time is too long, the system is likely to be lost with the lost fluid and unable to stay and form a gel at the target position, ultimately resulting in decreased plugging efficiency and poor lost-circulation control effect.

**Table 3:** Gel-strength code, adopted directly from Reference [32] without modification.

Strength Code	Gel Name	Corresponding Strength Description
A	Non-detectable gel	The viscosity of the system is comparable to that of the polymer solution, and no gel formation is visible to the naked eye.
B	Highly flowable gel	The viscosity of the gel system is slightly higher than that of the polymer solution.
C	Flowable gel	Most of the gel can flow to the other end of the vial when inverted.
D	Moderately flowable gel	A small fraction of the gel (<15%) cannot flow to the other end upon inversion and usually exists as a tongue-like protrusion.
E	Almost non-flowable gel	A small amount of gel can slowly flow to the other end upon inversion, while the majority (>15%) is non-flowable.
F	Highly deformable non-flowable gel	The gel cannot flow to the vial mouth when inverted.
G	Moderately deformable non-flowable gel	The gel can only flow to the middle of the vial upon inversion.
H	Slightly deformable non-flowable gel	Only the gel surface deforms upon inversion.
I	Rigid gel	No deformation of the gel surface occurs upon inversion.
J	Rattler gel	A tuning-fork-like mechanical vibration can be sensed when the vial is shaken.

### 4.6. Determination of Gelation Time

The gelation time was determined using the visual scoring method [33], defined as the time required for the system to transform from an initially flowable solution (code A) into a rigid gel (code I). Experiments in each group were performed in triplicate. However, the gel prepared in this study is a viscoelastic system with soft texture and high overall viscosity. Limited by the properties of the crosslinking system, the final strength of the system cannot reach the rigid gel level corresponding to code I. Moreover, when the system reaches the state of code G (moderately deformable non-flowable gel), it completely loses fluidity and can meet the engineering requirements of formation plugging. Therefore, in this study, the gelation time is defined as the time required for the system to transform from an initially flowable solution (code A) into a moderately deformable non-flowable gel (code G).

In the experiment, the uniformly mixed delayed crosslinking system was loaded into sealed test tubes and aged isothermally at a set temperature. The state of flow was observed by inverting the tubes periodically. The time at which the system maintained its shape (code G) was recorded as the gelation time, thereby characterizing the delayed crosslinking performance of the system.

### 4.7. Determination of Temperature Resistance of the Gel

The temperature resistance of the gel was measured using a HAAKE rheometer (Model HAAKE MARS 60, Thermo Fisher Scientific Inc., Karlsruhe, Germany), with the elastic modulus (G′) and viscous modulus (G″) as evaluation indicators. Dynamic oscillation tests were carried out at four temperatures: 30 °C, 60 °C, 90 °C, and 120 °C. The detailed procedures are as follows: The gel was first cut into uniform blocks with a diameter of approximately 20 mm and a thickness of 1–2 mm, ensuring a flat and intact surface. Three parallel samples were prepared and placed in clean Petri dishes for later use. Subsequently, the rheometer was turned on and preheated for 30 min. A 50 mm parallel-plate fixture was selected, and the gap was adjusted to 1 mm. Calibrations of temperature, torque, and strain were completed with an error ≤±2%. The linear viscoelastic region was determined by amplitude sweep, and 1% strain was chosen as the test parameter. The sample was placed at the center of the fixture, and the excess gel was trimmed off. Silicone oil was applied around the fixture edge to prevent water loss, and a thermal cover was placed to reduce temperature fluctuations. Dynamic temperature-step scanning was performed at the four set temperatures in sequence. Each temperature was held for 10 min to ensure thermal equilibrium between the sample and the test environment.

### 4.8. Evaluation of Plugging Strength

**Figure 14:**
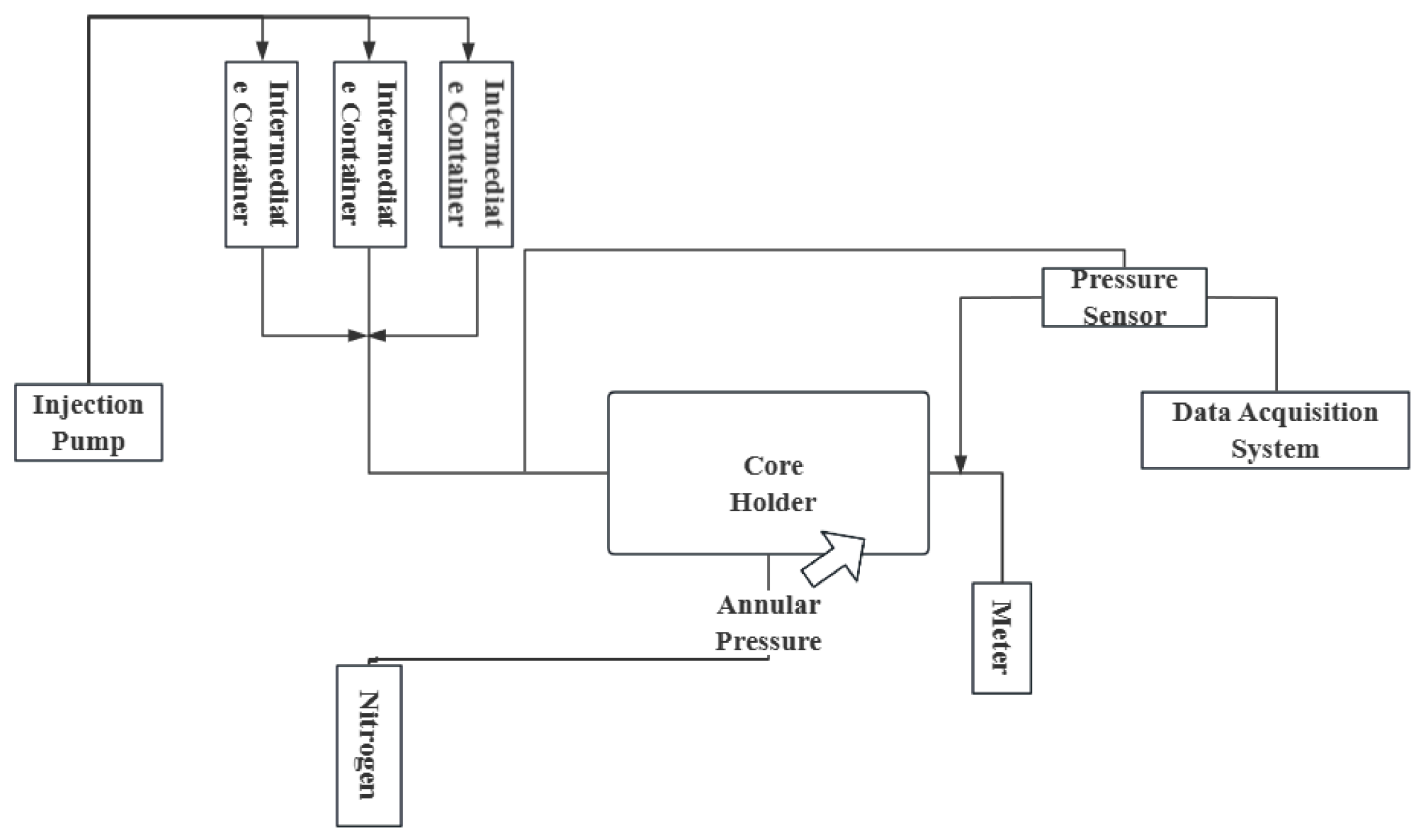
Displacement experimental setup.

The plugging performance of the gel was evaluated using a displacement experimental setup, as shown in Figure 14. Quartz sand-packed tubes were used to replace fractured cores, and quartz sands of different mesh sizes were selected to prepare the packed tubes, so as to simulate the characteristics of formation fractures and high-permeability channels. After vacuum saturation with simulated formation water, pressure was applied by injecting water from one end of the core holder using a constant-flow pump. After the inlet and outlet pressures and the outlet flow rate became stable, the displacement flow rate, pressure difference between the two ends, and geometric parameters of the sand-packed tube were recorded. The water-measured permeability *K*_0_ of the sand-packed tube was calculated according to Darcy’s law (6).

(6) $$K_{0}=\frac{Q\cdot \mu \cdot L}{A\cdot \Delta P}\times 0.1$$

In this formula, *K*_0_—initial permeability, D;

*q*—fluid flow rate, cm^3^·s^−1^;

*μ*—viscosity of the injected fluid, mPa·s;

*L*—effective length of the core, cm;

*A*—cross-sectional area of the core, cm^2^

Δ*P*—pressure difference across the core, MPa.

The gel was injected into the sand-packed tube at the same flow rate with an injection volume of 1.0–1.5 pore volumes (PV). The pressure variation during injection was recorded in real time. After injection, the valves at both ends of the core holder were closed, and the sand tube was kept static at the simulated formation temperature for a certain period to ensure sufficient gelation. The static aging time was set to 4–8 h according to the gelling characteristics of the gel. After completion of gelation, simulated formation water was injected again at the same flow rate for post-water flooding. When water flowed out from the other end, the pressure value at this moment was recorded as the breakthrough pressure. After the pressure was stabilized again, the permeability after plugging *K*_1_ and plugging efficiency *E* was calculated. Experiments in each group were performed in triplicate.

(7) $$E=\frac{K_{0}-K_{1}}{\;K_{0}}\times 100\%$$

In this formula, E —plugging efficiency, %;

*K*_0_—initial water permeability, mD;

*K*_1_—permeability after gel plugging, mD.

## Figures and Tables

**Table 1:** Plugging performance parameters of gel at different temperatures and quartz sand mesh sizes.

Temperature/°C	Quartz Sand/Mesh	Initial Permeability K_0_/D	Breakthrough Pressure P/MPa	Permeability After Gel Plugging K_1_/D	Plugging Efficiency E/%
	10	8.200 ± 0.100	5.150 ± 0.050	0.783 ± 0.153	90.45 ± 1.79
30	20	4.207 ± 0.110	5.480 ± 0.053	0.360 ± 0.053	91.42 ± 1.46
	40	2.853 ± 0.045	6.223 ± 0.093	0.247 ± 0.055	91.36 ± 1.88
	10	8.370 ± 0.072	5.423 ± 0.055	0.660 ± 0.040	92.80 ± 0.50
60	20	4.207 ± 0.090	5.967 ± 0.042	0.233 ± 0.045	94.44 ± 1.12
	40	2.950 ± 0.050	6.517 ± 0.076	0.143 ± 0.021	95.15 ± 0.66
	10	8.417 ± 0.236	5.133 ± 0.208	0.967 ± 0.153	88.54 ± 1.57
90	20	4.393 ± 0.181	5.093 ± 0.101	0.400 ± 0.044	90.86 ± 1.38
	40	2.827 ± 0.064	5.433 ± 0.126	0.267 ± 0.015	90.56 ± 0.73

**Table 2:** Comparative performance of delayed-crosslinking plugging systems under matched conditions (70 °C, HPAM 2000 mg/L, salinity 5000 mg/L NaCl).

System	Delay Time (to Gelation)	Temp. Range (°C)	Breakthrough Pressure (MPa/m)	Relative Cost
Ligand-shielded Zr^4+^	6–8 d	≤90	1.8–2.5	High
Ligand-shielded Cr^3+^ (acetate)	10–15 d	≤80	2.0–3.1	Medium
Redox Cr^6+^/Cr^3+^	2–48 h (poorly controllable)	≤70	1.5–2.0	Low
Microcapsule-coated Cr^3+^	16–32 h (tunable by coating thickness)	≤80	1.8–2.5	High
ZnO-supported system (this work)	20–50 h (tunable)	≤120	2.8–3.5	Low

## Data Availability

All data, models, and codes generated or used during the study are Available from the corresponding author by request.
